# Optimizing the Delivery of Routine Malaria Data Quality Assessments: A Two-Level Logistic Regression Model to Inform an Institutionalized Approach in Mozambique

**DOI:** 10.4269/ajtmh.23-0443

**Published:** 2024-09-24

**Authors:** Ann-Sophie Stratil, Maria Rodrigues, Sarmento Armando, Sergio Gomane, Kulssum Mussa, Baltazar Candrinho, Arantxa Roca-Feltrer

**Affiliations:** ^1^ Malaria Consortium, London, United Kingdom;; ^2^ Malaria Consortium, Maputo, Mozambique;; ^3^ National Malaria Control Programme at Ministry of Health, Maputo, Mozambique

## Abstract

Mozambique has implemented routine data quality assessments (DQAs) to improve accuracy of health facility (HF) malaria reporting since 2019. However, despite this being a resource-intensive exercise, the impact of operational factors on DQAs has not yet been systematically investigated. This analysis aimed to provide insights into optimizing the operational delivery of routine DQAs. A two-level logistic regression model based on 1,354 DQAs conducted across 195 HFs (16 districts, November 2019–December 2022) was used to estimate the impact of relevant operational factors, namely number of DQAs received, baseline reporting accuracy, HF setting, workload, malaria transmission intensity, and the shift to digital reporting, on accurate reporting by HFs. A report was considered accurate if the deviation between number of confirmed malaria cases in reports and register books was <10%. A statistically significant interaction was observed between baseline reporting accuracy and number of DQAs. For HFs with a baseline accuracy of ≤90%, each additional DQA increased the odds of accurate reporting by 102.8% (95% CI: 71.1–140.2%). For HFs with inaccurate data at baseline, the probability of accurate reporting increased to >80% after five DQAs, whereas HFs with accurate baseline data did not improve beyond the baseline visit. Other operational factors did not significantly affect reporting accuracy. Prioritizing HFs with low baseline accuracy for more frequent DQAs (every 6 months) with at least one visit to all HFs every 3 years might optimize resource allocation in Mozambique. Similar analytic approaches can be applied in other countries to optimize resource allocations for the delivery of routine DQAs.

## INTRODUCTION

Robust systems for capturing malaria data are essential for effectively monitoring progress toward malaria targets and are therefore central in facilitating data-informed decisions and enabling governments to prioritize and allocate necessary resources.^1^ However, despite national programs collecting data on routine health services, data often remain underutilized owing to concerns regarding their poor quality, leading to a lack of trust in their accuracy and reliability.^2^ Fewer than one in five countries reported having comprehensive systems of documented quality checks for facility data in both primary care facilities and hospitals.^3^ To address this critical gap, a significant number of routine malaria data quality assessment (DQA) tools have been developed over the last years to define data quality standards and harmonize implementation efforts by partners and national programs.^4^^–^^7^ These tools still require adaptation to country contexts, leading to high variability of methodology, scope, and scale on how DQAs are implemented operationally.

Despite the very limited documented evidence of routine DQAs on data quality improvements, particularly for malaria, several studies showed that implementing regular DQAs contributed positively to data accuracy.^8^^,^^9^ However, no study has specifically looked at the methodological factors that influence improvements in accuracy over time when implementing routine malaria DQA activities. These include, for example, the minimum frequency of DQAs per health facility (HF) to achieve a targeted accuracy threshold and the influence of baseline accuracy, as well as the effect of HF setting, HF workload, and malaria transmission intensity. Documentation of the impact of DQAs at scale on improvements in accuracy of malaria data, as well as the influence of key methodological aspects, is therefore needed to optimize the institutionalization of routine malaria DQAs and the use of programmatic resources to maximize the impact of these core surveillance-strengthening activities.

**Figure 1.**
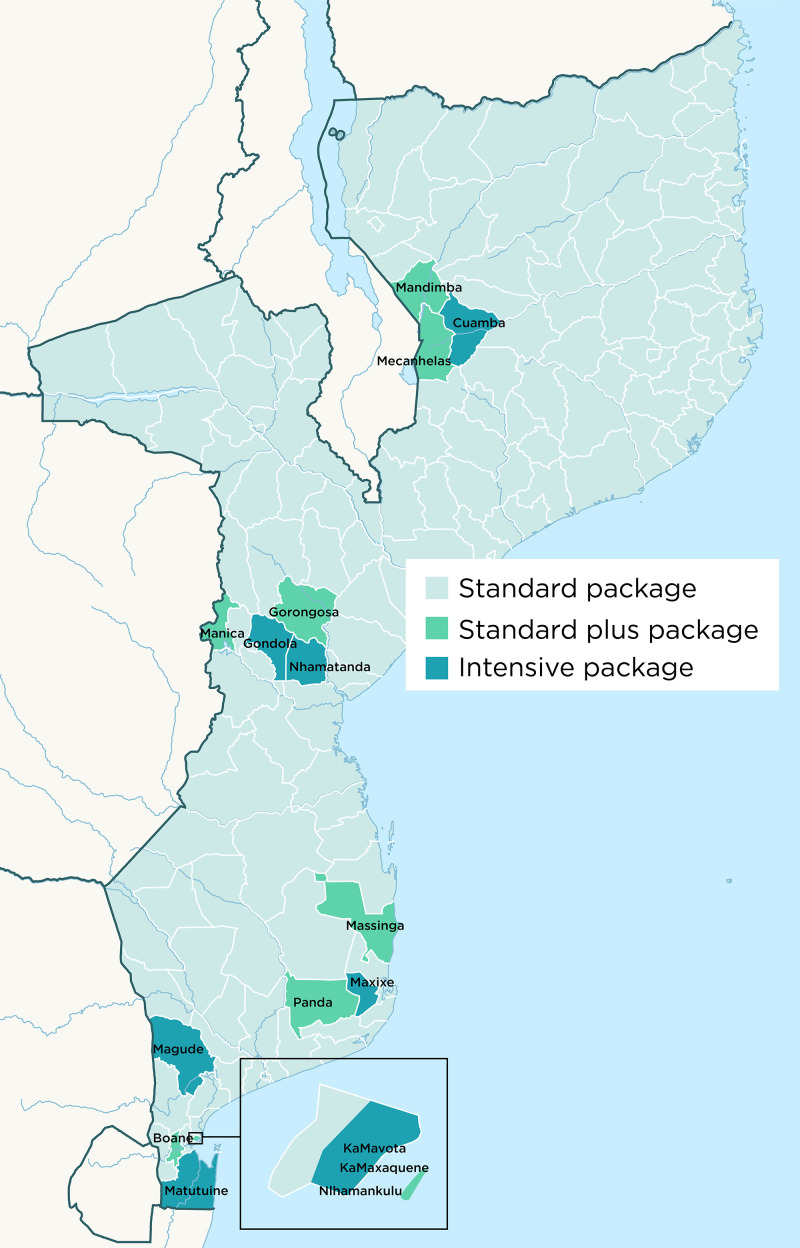
Map of selected districts by intervention package.

The National Malaria Control Program (NMCP) in Mozambique prioritized data-driven decision-making in its National Malaria Control Strategic Plan 2017–2022.^10^ Under the leadership of the Mozambique NMCP, a 3-year surveillance-strengthening project to guide optimal operationalization of surveillance activities and embed them into day-to-day data reporting, data use, and data-to-action activities was implemented between May 2019 and December 2022. Three different surveillance intervention packages were implemented: a standard intervention package nationally, a standard plus package in 11 districts, and an intensive package in five focus districts, each receiving a different set of interventions (see Figure 1). Apart from implementing routine malaria DQAs at the HF level, interventions also included rolling out supportive supervision of community health workers, HF, and district malaria focal points (DNFPs); procuring and distributing malaria recording and archiving forms; deploying an integrated malaria information storage system (iMISS) at district and HF levels; and introducing digital collection of aggregated data at the HF level.^11^ Table 1 provides an overview of interventions related to improving data quality implemented in each package. As this was the first time Mozambique introduced DQAs at the HF level nationally, a manual outlining the national approach to malaria DQAs and the operationalization of DQA indicators was published in February 2019 and followed by nationwide training of relevant district-level staff.^12^

**Table 1:** Interventions related to DQAs by intervention package

Interventions	Intervention Package		
	Standard	Standard Plus	Intensive
Electronic DQA Reporting and Analyses	x	x	x
DQA Training and Supervision Guidance and Support	x	x	x
Printing and Distribution of Essential Routine Reporting Tools in Targeted Districts	x	x	x
Roll-Out of Integrated Malaria Storage System at District Level	x	x	x
M&E, Data Use, and Data-to-Action Guidance, Development, and Training	x	x	x
Post-DQA Training–Tailored Supervision for District and HF Staff	–	x	x
Supportive Supervision at District and HFs Focused on Data Quality and Follow-Up Actions	–	x	x
Remote (central/provincial) Level Support, Mentoring and Coaching for Data DQA, Data Use, and Data to Action	–	x	x
Sharing of Experiences across Neighboring Districts, HFs, through Participation in DQA, Data Use and Data-to-Action Activities, and Data Review Meetings	–	x	x
Roll-Out of Integrated Malaria Storage System at HF Level	–	–	x
Support of DQ, Data Use, and Data-to-Action Follow-Up at HF Level, and Data Quality Competitions and Bulletins	–	–	x

DQ = data quality; DQA = data quality assessment; HF = health facility; M&E = monitoring and evaluation.

This analysis used the data collected through routine DQA activities with the aim of assessing the influence of different operational factors on the impact of DQAs on improved accuracy of HF malaria data and determine the optimal number of routine DQAs that HFs should receive to improve performance.

## MATERIALS AND METHODS

### Analysis design and setting.

This retrospective analysis examined longitudinal data to assess the impact of routine DQAs on the accuracy of malaria monthly reports compared with register books reported by HFs in Mozambique. The analysis used data collected during routine DQAs conducted between November 2019 and December 2022.

Each month, malaria focal points at HFs fill in monthly malaria reports where they aggregate key malaria variables extracted from register books that contain patient-level information. Key malaria variables include the number of consultations, number of microscopy and rapid diagnostic tests (RDTs) conducted, number of clinical malaria cases detected, and number of malaria cases confirmed by microscopy or RDT as well as the number of malaria cases treated. These malaria reports are submitted to the district level and entered into the central database DHIS2 by district malaria focal points DMFPs. From November 2019, DMFPs conducted routine DQAs at HFs in adherence with the national DQA manual to assess the data quality of monthly malaria reports. The DQAs were followed up by action plans that listed concrete actions for HFs or district or province level, depending on the action identified, and were monitored for compliance. In addition, monthly data review meetings took place at the district level with participation of HF focal points after DQAs. Compliance to action plans and discussion points at monthly review meetings were not systematically collected and could therefore not be considered in the analysis.

### Data collection.

During each DQA, DMFPs extracted key malaria variables from HF register books and monthly malaria reports. Data were collected using standardized paper-based forms. During the evaluation period, there was no systematic data repository available for DQA data yet. Hence, forms were forwarded to data entry clerks who entered the data into an Excel-based database and verified data consistency.

### Sample.

This analysis included all HFs in the 16 standard plus and intensive package districts. Data from HFs in the standard package were not included, as the conduction of routine DQAs in the standard package districts suffered from delays owing to reprioritization because of COVID and lack of a systematic mechanism for data reporting. The standard plus (*N* = 103) and intensive package (*N* = 94) districts comprised a total of 197 HFs, which represented 10.7% of the total 1,841 HFs in Mozambique. Only HFs that had received more than one DQA by the end of the evaluation period were included in the analysis to allow analysis of impacts over time.

### Variables.

To estimate accuracy, this analysis focused on malaria cases confirmed by RDT or microscopy, as this variable was considered the most critical for understanding malaria trends and impacting data-informed decision-making. Accuracy was calculated as the difference between the number of malaria cases confirmed by microscopy or RDTs in the monthly reports and register books over the number of confirmed cases in register books. Because of the inability to differentiate between blanks (e.g., missing fields) and zeros (e.g., zero cases reported), a conservative imputation approach was used, treating all blanks as zeros. In instances where the number of confirmed cases in the register book was zero, the accuracy was recorded as 0% if there was a nonzero absolute difference between the register book and the report. Conversely, if the absolute difference was zero, the accuracy was inputted as 100%. A report contained accurate confirmed malaria case data if the deviation between reports and register books was <10% (i.e., showed an accuracy of >90%). This threshold was based on the national DQA manual.^12^

Operational factors were defined a priori: number of consecutive DQAs (continuous), baseline accuracy of confirmed malaria cases in monthly report (categorized as “inaccurate” or “accurate”), HF setting (categorized as “urban” or “rural” based on national health information system data), HF workload (categorized based on the mean number of consultations across DQAs: <700, 700–1,500, >1,500 consultations), malaria transmission intensity (categorized based on the mean proportion of confirmed positive cases among consultations: ≤5%, 6–25%, >25% positive), and shift to digital reporting of aggregated data at the HF level (yes, no, implemented in selected districts from February 2021). For HF workload and transmission intensity, categories were chosen to ensure an approximately equal distribution of HFs across groups. Roll-out of digital collection of aggregated data at the HF level was considered a potentially relevant factor, as it changed the process of how monthly indicators were reported to the central database. Distribution of register books was not considered, as all HFs had the same initial availability.

## STATISTICAL ANALYSES

To account for the hierarchical structure of our data, with HFs nested within districts, a two-level logistic regression model was used. This model estimated the probability of an individual HF reporting accurate confirmed malaria cases (categorized as inaccurate: ≤90% accuracy or accurate: >90% accuracy) in dependence of the operational factors described above. Operational factors included in the model were determined via a stepwise variable selection approach. We first conducted univariate logistic regression analyses for each predefined operational factor with reporting accuracy (categorized as inaccurate: ≤90% accuracy or accurate: >90% accuracy) as the dependent variable. Only factors with a statistically significant Wald test at *P <*0.05 were retained for further analysis. Next, we built a multivariable logistic regression model including all the significant factors identified in the previous step. In addition, we included an interaction term between the number of DQAs and baseline accuracy in this model to assess potential modification effects. The significance of the interaction term was evaluated using the likelihood ratio test with a significance level of *P <*0.05.

Data were analyzed using STATA 13 (Stata Statistical Software: Release 13, StataCorp LP, College Station, TX).

## RESULTS

### Sample description.

This analysis included data from a total of 1,354 DQAs conducted at 195 HFs located within 16 districts in Mozambique between November 2019 and December 2022. Two HFs were excluded from this analysis because they had received only one DQA throughout the analysis period owing to accessibility issues. Among the included sample of 195 HFs, 84.6% were located in rural areas. At baseline (first DQA), 48.2% of HFs exhibited inaccurate monthly reports compared with the register books; 63.3% of rural HFs and 45.5% of urban HFs had inaccurate monthly reports at baseline. On average, each HF received 4.1 (minimum 2 and maximum 10) DQAs over the 3-year-long evaluation period; 44.1% of HFs started to record monthly reports digitally from February 2021. Table 2 outlines baseline characteristics of included HFs.

**Table 2:** Characteristics of HFs by baseline accuracy of report

Baseline Accuracy	Inaccurate (≤90%)	Accurate (>90%)	Total
Number of HFs at Baseline	94 (48.2%)	101 (51.8%)	195 (100%)
Number of HFs in Rural/Urban Setting			
Rural Setting	75 (45.5%)	90 (54.6%)	165 (100%)
Urban Setting	19 (63.3%)	11 (36.7%)	30 (100%)
Number of HFs That Reported the Following Mean Number of Consultations per Month across DQAs (based on register books)			
<700	19 (41.3%)	27 (58.7%)	46 (100%)
700–1,500	41 (51.3%)	39 (48.8%)	80 (100%)
>1,500	34 (49.3%)	35 (50.7%)	69 (100%)
Number of HFs That Reported the Following Mean Proportion of Confirmed Cases among Consultations per Month across DQAs (based on register books)			
≤5%	20 (42.6%)	27 (57.5%)	47 (100%)
>5% to ≤25%	30 (41.1%)	43 (58.9%)	73 (100%)
>25%	44 (58.7%)	31 (41.3%)	75 (100%)
Number of HFs That Digitally Reported Aggregated Data at HF Level from February 2021			
Yes	45 (52.3%)	41 (47.7%)	86 (100%)
No	49 (45.0%)	60 (55.1%)	109 (100%)
Number of DQAs Conducted over Evaluation Period	671 (49.6%)	683 (50.4%)	1,354 (100%)
Average Number of DQAs Received over Evaluation Period, Mean (minimum-maximum)	4.1 (3–10)	4.2 (2–10)	4.1 (2–10)

DQA = data quality assessment; HF = health facility.

### Impact of DQAs.

**Figure 2.**
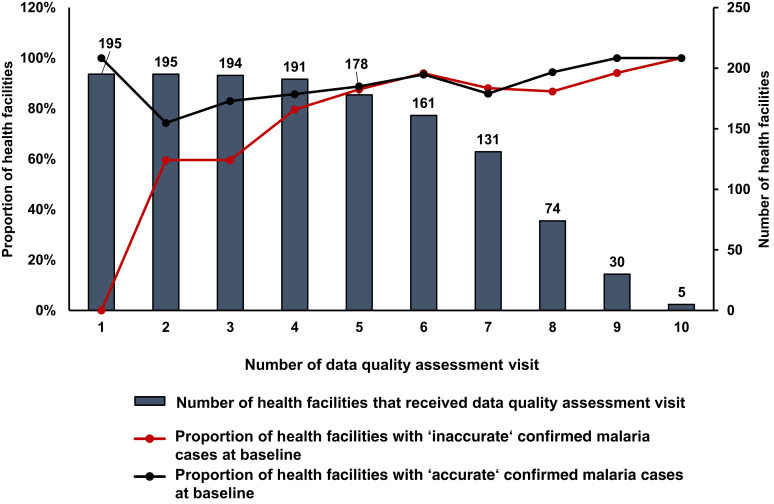
Frequency of accurate and inaccurate reporting. Proportion of health facilities reporting accurate confirmed malaria cases by data quality assessment visit and baseline accuracy.

At baseline, 48.2% of HFs had reports with inaccurate confirmed malaria cases compared with the register books. At the last DQA, 93.1% of HFs had an accurate report. Between 28.7% and 81.0% of the HFs that had a report with inaccurate confirmed malaria cases at the previous visit had transitioned to having a report with accurate confirmed malaria cases at the consecutive one. Table 3 outlines results by DQA. The proportion of HFs with accurate reports steadily increased with each additional DQA (Figure 2).

**Table 3:** Proportion of health facilities that reported accurate confirmed malaria cases by DQA visit and baseline accuracy of report

Baseline Accuracy	Inaccurate Confirmed Malaria Cases (≤90%)	Accurate Confirmed Malaria Cases (>90%)	Total
Number of HFs That Had Accurate Reports at the DQA Last Visit			
Yes	85 (90.4%)	94 (93.1%)	179 (91.8%)
No	9 (9.6%)	7 (6.9%)	16 (8.2%)
Number of HFs That Had Accurate Reports at Each Respective Visit			
1	0/94 (0%)	101/101 (100%)	101/195 (51.8%)
2	56/94 (59.6%)	75/101 (74.3%)	131/195 (67.2%)
3	56/94 (59.6%)	83/100 (83%)	139/194 (71.6%)
4	74/93 (79.6%)	84/98 (85.7%)	158/191 (82.7%)
5	78/89 (87.6%)	79/89 (88.8%)	157/178 (88.2%)
6	79/84 (94%)	72/77 (93.5%)	151/161 (93.8%)
7	59/67 (88.1%)	55/64 (85.9%)	114/131 (87%)
8	33/38 (86.8%)	34/36 (94.4%)	67/74 (90.5%)
9	16/17 (94.1%)	13/13 (100%)	29/30 (96.7%)
10	1/1 (100%)	4/4 (100%)	5/5 (100%)
Number of HFs with Reports with Inaccurate Confirmed Malaria Cases That Had Accurate Report Data at the Next Visit			
1	56/94 (59.6%)	0/94 (0%)	56/94 (59.6%)
2	18/64 (28.1%)	21/64 (32.8%)	39/64 (60.9%)
3	27/55 (49.1%)	13/55 (23.6%)	40/55 (72.7%)
4	13/33 (39.4%)	10/33 (30.3%)	23/33 (69.7%)
5	11/21 (52.4%)	6/21 (28.6%)	17/21 (81.0%)
6	4/10 (40%)	3/10 (30%)	7/10 (70%)
7	2/17 (11.8%)	6/17 (35.3%)	8/17 (47.1%)
8	2/7 (28.6%)	0/7 (0%)	2/7 (28.6%)
9	–	–	–

DQA = data quality assessment; HF = health facility.

**Figure 3.**
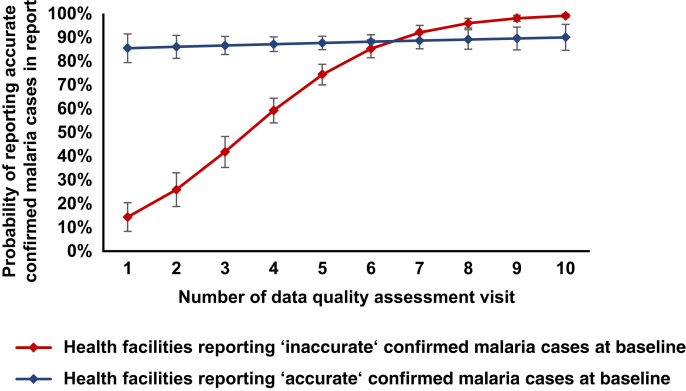
Predicted probabilities of accurate reporting. Expected probabilities of reporting accurate confirmed malaria cases by data quality assessment visit stratified by baseline accuracy (95% CI).

Univariate analysis using a two-level logistic regression model identified significant effects (Wald test, *P <*0.05) between predefined operational factors (number of DQAs, baseline accuracy, HF setting, HF workload, and shift to digital reporting) and accurate reporting. All these factors were included in the final model. The proxy for malaria transmission intensity had no significant effect on accurate reporting (Wald test, *P* >0.05) and was excluded from the final model. The analysis further identified a significant interaction effect (Likelihood-Ratio test, *P <*0.05) between the number of DQAs and baseline accuracy. In the initial model without the interaction term, only baseline accuracy and number of DQAs significantly impacted accurate reporting. Each additional DQA increased the odds of accurate reporting by 54.3% (95% CI: 40.9–69.1%). In addition, compared with HFs with accurate baseline reports, HFs with inaccurate baseline reports had approximately 4.29 times lower odds (95% CI: 3.05–6.05) of having an accurate report. However, accounting for the interaction effect of baseline accuracy on the association between number of DQAs and accurate reporting revealed a modifying effect. For HFs with inaccurate baseline reporting, each additional DQA increased the odds of accurate reporting by 102.8% (95% CI: 71.1–140.2%). For HFs with inaccurate data at baseline, the probability of reporting accurate confirmed malaria cases steadily increased to >80% after five DQAs, whereas for HFs with accurate data at baseline, the same probability remained approximately 80% for each visit (Table 3; Figure 3). Other operational factors did not significantly affect the odds of reporting accurate data (*P* ≥0.05).

## DISCUSSION

This is the first time that the impact of routine DQAs on the accuracy of HF monthly malaria reports was quantified in relation to various operational factors. Results showed that each additional routine DQA had a significant positive effect on accuracy of monthly malaria reports when baseline reporting was inaccurate.

Other studies have previously shown similar positive effects of routine DQAs on the accuracy of routine data. An assessment of routine DQAs of electronic medical records in 341 HFs in Kenya showed that concordance between data in register books and electronic medical records meaningfully improved after one DQA.^8^ A study across 444 HFs in Sierra Leone showed that four DQAs delivered over 2 years led to significant improvement in the proportion of accurate reports on the Integrated Disease Surveillance and Response System by 19.5% (95% CI: 12.5–26.5%).^13^ The same study also found that improvements in data accuracy stagnated after the first visit. This might be due to not accounting for baseline accuracy, which was shown to be a modifying factor in the current evaluation. Our analysis showed that for HFs with inaccurate confirmed malaria cases at baseline, the probability of reporting accurate confirmed malaria cases steadily increased to over 80% after five DQAs, whereas for HFs where confirmed malaria case data were already accurate, the same probability did not further improve (Table 4). Our findings translate to an optimal DQA frequency of one DQA every 6 months if the HF reported inaccurate confirmed malaria cases at the initial baseline visit, based on the assumption that equal spacing between DQAs is optimal. Every 3 years, a rapid DQA to all HFs that did not receive ongoing DQAs (as they had reported accurate confirmed malaria cases at the initial baseline visit) could be useful to reassess baseline accuracy to prioritize efforts and resources in subsequent DQAs. This exercise could also be linked to a broader assessment of the malaria surveillance system, including tools created by the WHO. The recommendation derived from the current analysis is similar to the guideline in the current Mozambique malaria DQA manual, which suggests that district focal points visit all HFs annually, with two further follow-up visits to HFs showing low–data quality performance.^12^ To ensure this time-intensive exercise is done in the most cost-effective way, we suggest focusing on the accuracy of one key data element (e.g., confirmed malaria cases), as it suffices as a proxy for general malaria accuracy at a given HF despite not measuring the data quality of all elements or quality dimensions (e.g., completeness and timeliness).

**Table 4:** Predicted probability of reporting accurate confirmed malaria cases by DQA visit

Number of DQAs	Predicted Probability of Reporting Accurate Confirmed Malaria Cases, % (95% CI)		
	Baseline Report with Inaccurate Number of Confirmed Malaria Cases	Baseline Report with Accurate Number of Confirmed Malaria Cases	Total
1	14.5 (8.4–20.5%)	85.3 (79.3–91.4%)	50.7 (46.2–55.1%)
2	26.1 (19.0–33.1%)	85.9 (81.1–90.8%)	56.6 (52.2–61.0%)
3	42.0 (35.5–48.5%)	86.5 (82.7–90.3%)	64.6 (60.8–68.4%)
4	59.4 (54.2–64.5%)	87.0 (83.9–90.1%)	73.3 (70.4–76.3%)
5	74.5 (70.2–78.8%)	87.6 (84.7–90.4%)	81.0 (78.4–83.6%)
6	85.3 (81.6–89.0%)	88.1 (85.1–91.2%)	86.7 (84.2–89.1%)
7	92.1 (89.1–95.0%)	88.6 (85.1–92.1%)	90.4 (88.0–92.8%)
8	96.0 (93.9–98.0%)	89.1 (85.0–93.2%)	92.7 (90.3–95.1%)
9	98.0 (96.7–99.3%)	89.5 (84.7–94.3%)	94.0 (91.6–96.5%)
10	99.0 (98.3–99.8%)	90.0 (84.5–95.4%)	94.8 (92.1–97.5%)

DQA = data quality assessment.

It can be argued that other factors may have influenced the data accuracy of malaria reports beyond routine DQAs. These include regular supportive supervision visits and data quality reviews, training and experience of the workforce, implementation of action plans, stock-outs of register books, and the shift to electronic reporting, as they were introduced simultaneously with routine DQA activities from the midpoint of the evaluation period (February 2021). For the current analysis, nationwide distribution of register books took place before the start of DQAs, and stock-outs were monitored throughout the evaluation period and were not reported as an issue during the visits. The introduction of electronic reporting of monthly data did not show any significant impact on data accuracy in the current evaluation. This seems reasonable, as the effect of electronic reporting on the accuracy of monthly malaria reports to the central database would be expected to show at the central database level, whereas the current evaluation focused on the comparison between monthly malaria reports and register books.^13^ Another study assessing the effect of annual DQAs across 26 HFs in Mozambique showed that the median concordance between register books and four key variables on the national health information system increased by 31.2% after 3 years.^9^ The same study also showed that HFs with more human resources made better improvements than HFs with fewer human resources.^9^ Owing to limited data availability, this evaluation did not include the influence of human resource shortages or turnover of key personnel or the reception of other surveillance-strengthening activities such as supportive supervision exercises and follow-up of action plans. Although the malaria workload was accounted for in the model through the proxy variable mean number of consultations across DQAs, it did not include a proxy for human resources. Further analyses might be needed to account for the influence of human resources, supportive supervision, and other mentioned factors on the impact of DQAs.

In Mozambique, key performance indicators from DQAs, including accuracy of monthly malaria reports, will be integrated into the iMISS, which was rolled out nationwide in 2021 and compiles malaria-related data from different routine systems. Information is available to all national and district staff as well as HF staff in selected districts and is visualized through tables and charts. Automated analysis will further improve the efficiency of assessing results from DQAs and help the timely identification of data quality problems at the HF level and subsequent allocation of appropriate resources. Continuous analyses of DQA findings should be embedded in DHIS2 analytics to help optimize delivery of DQAs and prioritize allocation of this key but resource-intensive activity. Further work looking at sensitivity analyses exploring other key variables and indicators and looking at the potential effect of incorporating missing values is needed for future refinements on routine Mozambique data quality analyses.

## CONCLUSION

This is the first time the impact of institutionalizing routine DQAs of accuracy on malaria data on routine reports was quantified in relation to various operational factors. Results showed that routine DQAs to HFs significantly improved the accuracy of routine malaria reports when baseline accuracy was low. Resources in Mozambique should prioritize HFs that have reports with inaccurate confirmed malaria cases with a suggested frequency of DQAs every 6 months and at least one DQA to all HFs every 3 years. Similar analytic approaches will help to optimize resource allocations for the delivery of routine DQAs in other countries.

## Data Availability

The data that support the findings of this study are available from the corresponding author upon reasonable request.
